# Impact of moderate-to-high-suicide-intent in major depressive disorder: a retrospective cohort study on patient characteristics and healthcare resource utilisation in England

**DOI:** 10.1186/s12888-024-05961-3

**Published:** 2024-08-23

**Authors:** Tom Denee, Cicely Kerr, Sarah Richards, Natalie Dennis, Astrid Foix-Colonier, Claire Fischer, Fintan Larkin

**Affiliations:** 1 Janssen-Cilag Ltd, 50-100 Holmers Farm Way, High Wycombe, Buckinghamshire, HP12 4EG UK; 2 Amaris Health Economics and Market Access, 7 Rue de Châteaudun, Paris, 75009 France; 3 Acute Mental Health Services, West London NHS Trust, London, UK

**Keywords:** Major depressive disorder, Suicide intent, Population-based study, England, Healthcare Resource use, Clinical practice research datalink (CPRD), Hospital episode statistics (HES), Mental health services data set (MHSDS), Office for National statistics (ONS)

## Abstract

**Background:**

Major depressive disorder (MDD) is a disabling mental illness that can affect all aspects of daily life and is a leading cause of healthcare resource utilisation (HCRU).

**Aims:**

We aimed to characterise patients with MDD with moderate-to-high-suicide-intent, compare their HCRU to patients with MDD without moderate-to-high-suicide-intent, and better understand their patient pathways.

**Methods:**

This retrospective cohort study used data collected from primary care electronic health records from Clinical Practice Research Datalink (CPRD), linked to Hospital Episode Statistics, Mental Health Services Data Set, and Office for National Statistics in England. Adults diagnosed with ≥ 1 MDD diagnosis between 04/2007 and 11/2015 were categorised by suicide intent.

**Results:**

307,476 patients with MDD were included (294,259 patients without moderate-to-high-suicide-intent and 13,217 with moderate-to-high-suicide-intent). Patients with MDD with moderate-to-high-suicide-intent were younger on average (39.0 vs. 44.8 years) and included a lower percentage of females (58% vs. 65%) compared to patients without moderate-to-high-suicide-intent. HCRU was greater among patients with moderate-to-high-suicide-intent than patients without moderate-to-high-suicide-intent during the first follow-up year for general practitioner consultations (38.5 vs. 29.4), psychiatric outpatient visits (1.5 vs. 0.1), psychiatrist visits (3.6 vs. 0.3), emergency visits (1.5 vs. 0.3), and hospitalisations (86% vs. 26%). Overall, 56% of patients with moderate-to-high-suicide-intent had an antidepressant prescription within 30 days from the initial moderate-to-high-suicide-intent.

**Conclusions:**

Patients with MDD and moderate-to-high-suicide-intent were younger, included more males and incurred greater HCRU than those without moderate-to-high-suicide-intent. These results suggest a greater need for effective medical care and appropriate treatments for patients with moderate-to-high-suicide-intent, which could help reduce associated symptoms, mortality, and HCRU.

**Supplementary Information:**

The online version contains supplementary material available at 10.1186/s12888-024-05961-3.

## Background

### Major depressive disorder in the United Kingdom

Major depressive disorder (MDD) is a disabling mental illness that can affect all aspects of daily life and is a leading cause of health-related economic costs [1, 2]. According to the Global Burden of Diseases, Injuries, and Risk Factors Study, depressive disorders accounted for 1.84% of total disability adjusted life years (DALYS) in 2019 [3]. Additionally, a disproportionate increase in the global burden of MDD in 2020 is seen due to the COVID-19 pandemic [4]. In a population-based study of depressive disorders in 27 European countries, the United Kingdom (UK) had the eight highest prevalence (7.40%) [5]. However, despite available interventions, no reduction in the global prevalence or burden of MDD has been documented since 1990 [6]. Antidepressants are part of MDD therapeutic landscape and have a key role to play in severe cases of depression: the association of individual cognitive behavioural therapy and antidepressant is the first treatment recommended by the National Institute for Health and Care Excellence (NICE) for more severe depression [7]. In clinical practice, patient response to antidepressant treatment often remains unsatisfactory, with 20–50% of MDD patients who had non-response from at least two antidepressant drug classes [8, 9]. Therefore, there is an urgent need for more effective antidepressant treatments for people with MDD.

### Suicidal ideation, a major symptom in MDD

Suicidal ideation is one of the core symptoms of MDD, which can develop into suicide intent and result in serious injury and early death. Although suicidal ideation is concerning and common, many sufferers from suicidal ideations have no intention to act on their suicidal thoughts. Patients with suicide intent have formed an intention to act on their suicidal thoughts, and represent a smaller, more vulnerable, and higher risk group of patients. One meta-analysis of observational studies showed a pooled prevalence of suicidal ideation of 37.7% and suicide planning of 15.1% in individuals with MDD [10].

Risk factors for suicide specific to depression include feelings of hopelessness, history of previous attempts including self-harm, anxiety, family history of mental illness, alcohol/drug abuse, and male gender [11]. Despite these known risk factors, a systematic review of six clinical practice guidelines (CPGs) for treatment of depression revealed that only 50% of CPGs included recommendations for the risk of suicide associated with pharmacological treatments [12].

The severity of suicide rates in patients with MDD was outlined by a recent meta-analysis, which found that patients with MDD presented the highest pooled suicide rate at 534.3 per 100,000 person-years, amongst patients with serious mental illness for whom the pooled rate was 312.8 per 100,000 person-years [13]. Studies on suicide rates specifically in the UK are scarce. The National Confidential Inquiry into Suicide and Safety in Mental Health (NCISH) database includes national case series of suicide by mental health patients over 20 years [14]. During 2007–2017, 13,806 deaths (27% of the general population suicides) were identified in people using mental health services in the 12 months prior to death, with affective disorders (bipolar disorder and depression) being the primary psychiatric diagnosis in 44% of the cases [14]. In a cohort study of 238,963 patients (20 to 64 years) with a first diagnosis of depression registered with UK general practices database, 198 cases of suicide and 5,243 cases of attempted suicide or self-harm occurred during the first year of follow-up [15].

While the aforementioned studies provide information on suicide rates among patients with MDD, the evidence on patients with MDD and moderate-to-high suicide intent is limited. Those patients have a higher risk of suicide and may require more intensive healthcare resources than other patients with MDD, but there are still significant gaps in understanding their characteristics and their healthcare pathway. Our study aims to address this need by characterising the healthcare resource utilisation (HCRU) of this vulnerable group, in England. A better understanding of the burden of disease is important for healthcare practices, identifying populations for innovative treatment development and related policy making. Indeed, it might encourage policies for better care of patients with MDD and moderate-to-high suicide intent, and to prevent the risk of developing moderate-to-high suicide intent among patients with MDD.

### Objectives

This study conducted in primary and secondary healthcare settings in England on patients with MDD with moderate-to-high-suicide-intent provides valuable data on this psychiatrically vulnerable group. The primary study objectives were to assess the clinical profile of patients with MDD and moderate-to-high-suicide-intent and to assess their HCRU compared to patients with MDD without suicide intent. Secondary objectives were to determine the incidence of moderate-to-high-suicide-intent, clinical outcomes, and treatment patterns. Findings of the study will help improve the understanding of patient characteristics, current management, HCRU, patient outcomes, and enhance clinical decision-making and policy development.

## Methods

### Study design and data source

This was a retrospective cohort study of adult patients diagnosed with MDD in England between April 2007 and November 2015, identified through healthcare data from four national sources: the Clinical Practice Research Datalink (CPRD) GOLD primary care database linked to Hospital Episode Statistics (HES) inpatient and outpatient data, the Mental Health Services Data Set (MHSDS), and the Office for National Statistics (ONS). The data were collected for this period as MHSDS was available only up to November 2015. The CPRD GOLD captures anonymised patient data from electronic healthcare records from a network of general practitioner (GP) practices across the UK, including conditions, observations, measurements, GP prescriptions, and procedures. HES monitors hospital activity and captures administrative data on all patient interactions at NHS hospitals in England. The MHSDS captures data on care services provided to individuals who use mental health services provided in hospitals, community settings, and outpatient clinics in England. ONS death registration dataset includes all deaths occurring in England and Wales. An eight-step deterministic algorithm encompassing NHS number, birth date, sex, and postcode (cf. Additional file 1, Table S2) was used to link eligible patients from those databases.

### Study population

This study included patients aged 18 years or older at index date who received an MDD diagnosis during the study period from a practice eligible for linkage to HES, MHSDS, and ONS. The Read and International Classification of Disease-10th revision (ICD-10) codes for MDD diagnosis (cf. Additional file 1, Table S3) have been used in a previous study of MDD and treatment resistant depression in England [16].

A list of suicide intent diagnoses (Read codes or ICD-10 code) was determined, and each diagnosis of the list was classified as “undefinable”, “moderate”, and “high” (cf. Additional file 1, Table S4-S5), with the help of clinical experts (F.L., V.M.). Diagnoses for suicide intent were then identified in CPRD (based on Read codes from GP consultations and referrals), HES (based on ICD-10 codes), and ONS (based on ICD-10 codes for cause of death).

Patients without a suicide intent diagnosis were eligible for inclusion in the MDD population. Patients with a “moderate” or “high” suicide-intent diagnosis before their first MDD diagnosis or in the 3 following months were eligible for inclusion in MDD and moderate-to-high-suicide-intent population. It was deemed that the three-month delay allowed patients whose MDD was first diagnosed following a moderate-to-high-suicide-intent to have time to be recorded as MDD patients in the data, as they already had a contact with a healthcare professional when the moderate-to-high-suicide-intent diagnosis was collected.

Patients with a diagnosis for psychosis, schizophrenia, mania, bipolar disorder, or dementia at any time in the history (before or after MDD diagnosis date) were excluded to mitigate any bias from including moderate-to-high-suicide-intent related to these conditions.

### Index date definition

For patients with MDD and moderate-to-high-suicide-intent, the index date is the date of the first moderate-to-high-suicide-intent diagnosis. Index date was required to be up to three months before or any time after the date of MDD diagnosis.

For patients with MDD without suicide intent, the index date corresponds to the first MDD diagnosis.

### Data collection and outcomes

For each patient, the following were evaluated: demographics (age, sex, region, and ethnicity) at the index date; body mass index (BMI), smoking status, Charlson Comorbidity Index (CCI) [17] within 12 months prior to the index date; incidence rate of moderate-to-high-suicide-intent (overall and by age and sex); presence of anxiety and substance abuse disorders in patients with at least one-year of follow-up; overall mortality rate, suicide-related mortality, mortality from other causes; HCRU and treatment patterns.

HCRU included GP consultations; Improving Access to Psychological Therapies (IAPT) services; psychiatric outpatient services by Crisis Resolution Home Treatment Team (CRHT) or Community Mental Health Team (CMHT); psychiatrist visits; accident and emergency visits; hospitalisations; psychiatric in-patient services; admissions to an intensive care unit (ICU) and psychiatric intensive care unit (psychiatric ICU), single point of access, 24/7 crisis response line, referrals to CMHT, IAPT, or single point of access (specific identification methods in Additional file 1, Table S6). In patients with MDD and without moderate-to-high-suicide-intent, HCRU was measured during the first follow-up year after the index date, and in patients with MDD and moderate-to-high-suicide-intent also during the first four weeks after the index date, amongst patients with appropriate follow-up duration. To avoid underestimating the burden by including patients with insufficient follow-up, the population used for each of these analyses was restricted to patients having a follow-up greater than or equal to the period of interest (number of patients included in each analysis in supplement, Table S1).

Data regarding treatment included pharmacological treatment (antidepressants alone or in combination with antipsychotics, anticonvulsants, or lithium on the same prescription date, or amitriptyline in doses > 100 mg/day, as lower doses may be prescribed for other indications such as pain management); interventional treatments (electroconvulsive therapy [ECT], transcranial magnetic stimulation [TMS], vagus nerve stimulation [VNS], and deep brain stimulation [DBS]); and non-pharmacological and/or non-interventional therapies (psychotherapy, occupational therapy, cognitive behavioural therapy, mindfulness, behavioural activation therapy, and health coach sessions).

The index pharmacological treatment was defined as drug treatment prescribed on or up to 30 days after the index date amongst patients with at least 30 days of follow-up. The subsequent treatment was the first treatment switch following the index treatment, with a change in combined treatment as a change in the line of therapy. Non-pharmacological/non-interventional therapies were assessed during the first follow-up year amongst patients with at least one-year of follow-up.

### Statistical analysis

The incidence of MDD with moderate-to-high-suicide-intent was calculated as the number of newly diagnosed patients with MDD and moderate-to-high-suicide-intent divided by the number of person-years at risk from April 2007 to November 2015. Newly diagnosed patients with MDD and moderate-to-high-suicide-intent consisted of patients whose moderate-to-high-suicide-intent was after MDD diagnosis (i.e. patients with moderate-to-high-suicide-intent before MDD diagnosis were excluded). After the initial MDD diagnosis, a patient was considered “at risk” of suicide intent until either first diagnosis of moderate-to-high-suicide-intent, end of the good quality follow-up period, or end of November 2015. The beginning of the good quality follow-up period was defined as the latest of the practice up-to-standard date, patient registration date and 1st April 2015. The end of the good quality period was defined as the earliest of the practice last collection date, patient transfer out date, and 30th November 2015.

The chi-squared test and Student’s t-test were used for the comparison of variables between patients with MDD with and without moderate-to-high-suicide-intent. Analyses were performed using SAS 9.4.

The switching of patients was modelled as a time-to-event outcome using Kaplan-Meier method, which enabled to compute the probability of switching.

## Results

### Patient characteristics

**Fig. 1:**
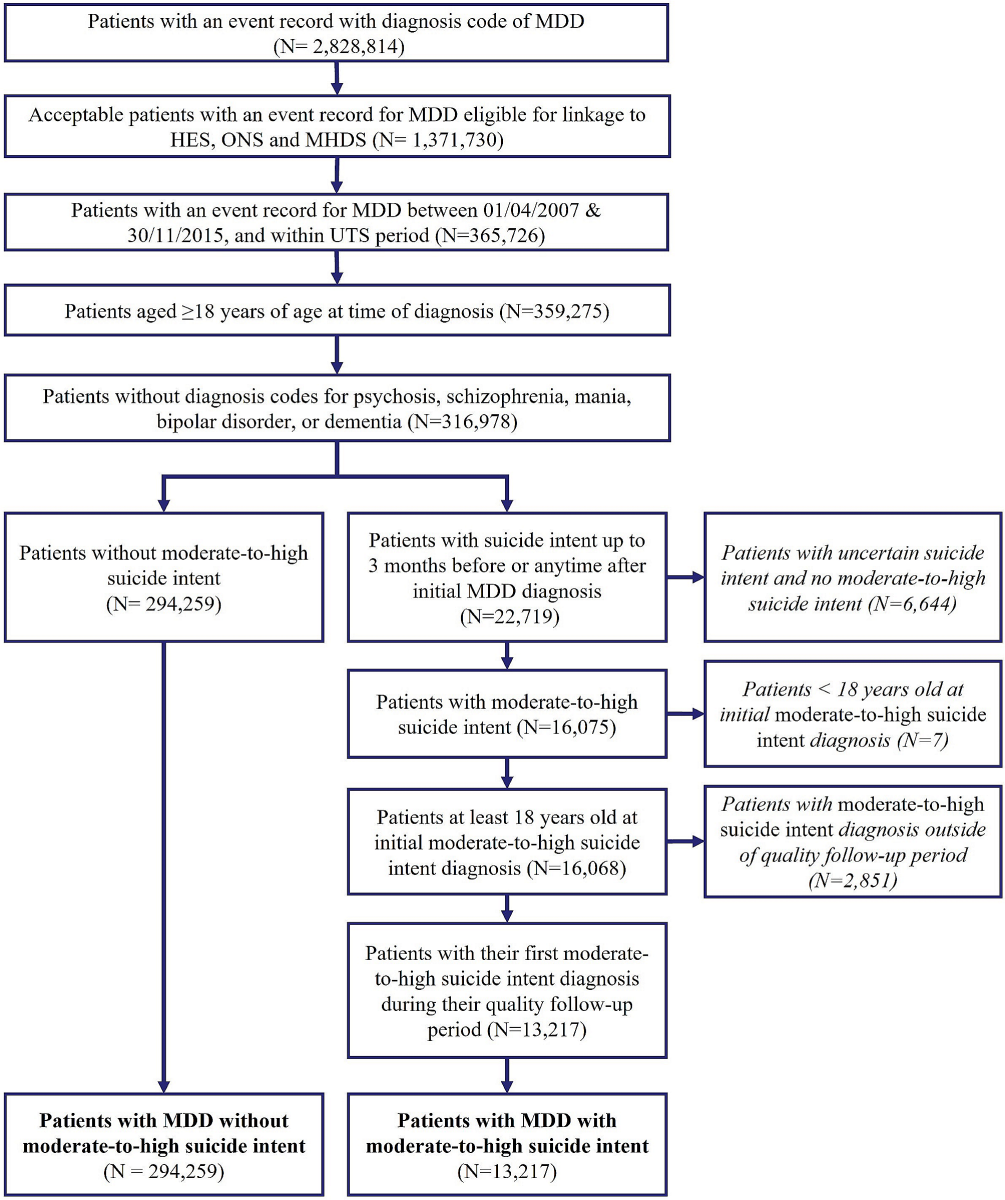
**Flow chart for the selection of patients included in the analysis**

During the study period, 307,476 patients were diagnosed with MDD. Among them, 13,217 were diagnosed with moderate-to-high-suicide-intent and 294,259 without suicide intent (Fig. 1). As shown in Table 1, the mean age was 44.5 years, and patients with MDD and moderate-to-high-suicide-intent were younger than those without suicide intent (39.0 vs. 44.8; *p* < 0.0001). More than 60% of patients were female, with a lower percentage of females in the group with moderate-to-high-suicide-intent (57.8% vs. 65.1%, *p* < 0.0001). Overall, the highest percentage of patients were from the North-West region (17.7% of patients), and the least represented region was the North-East (2.2% of patients). Hence, the distribution of patients was similar to that of the whole CPRD English population, except for the percentage in London, which was lower (11.3% vs. 17.9% in CPRD English population). Furthermore, most patients (88.2%) were white, particularly in the MDD and moderate-to-high-suicide-intent group (94%). More than 65% of the overall patient group had no comorbidities (CCI score = 0).

**Table 1:** Clinical characteristics of patients with major depressive disorder (MDD), overall and without and with moderate-to-high-suicide-intent

Variables	MDD overall (*n* = 307,476)	MDD without moderate- to-high suicide intent (*n* = 294,259)	MDD with moderate-to-high-suicide-intent (*n* = 13,217)	*P*-value*
**Age**,** years**, mean (SD)	44.5 (17.0)	44.8 (17.1)	39.0 (14.7)	< 0∙0001^a^
**Female**, N (%)	199,334 (64.8%)	191,692 (65.1%)	7,642 (57.8%)	< 0∙0001^b^
**Region**, N (%)				< 0∙0001^b^
East Midlands (0.9% of the English population in CPRD^†^)	7,019 (2.3%)	6,691 (2.3%)	328 (2.5%)	
East of England (9.1%)	29,963 (9.7%)	28,856 (9.8%)	1107 (8.4%)	
London (17.9%)	34,827 (11.3%)	33,711 (11.5%)	1116 (8.4%)	
North East (2.0%)	6,929 (2.2%)	6,518 (2.2%)	411 (3.1%)	
North West (15.5%)	54,477 (17.7%)	51,492 (17.5%)	2985 (22.6%)	
South Central (16.2%)	42,085 (13.7%)	40,202 (13.7%)	1883 (14.2%)	
South East Coast (14.1%)	46,171 (15.0%)	44,456 (15.1%)	1715 (13.0%)	
South West (11.2%)	42,054 (13.7%)	40,155 (13.6%)	1899 (14.4%)	
West Midlands (11.7%)	34,495 (11.2%)	33,065 (11.2%)	1430 (10.8%)	
Yorkshire & The Humber (1.4%)	9,456 (3.1%)	9,113 (3.1%)	343 (2.6%)	
**Ethnicity**, N (%)				< 0∙0001^b^
White	271,263 (88.2%)	258,846 (88%)	12,417 (94%)	
Asian	6,573 (2.1%)	6,399 (2.2%)	174 (1.3%)	
Black	3,989 (1.3%)	3,882 (1.3%)	107 (0.8%)	
Mixed	1,352 (0.4%)	1,289 (0.4%)	63 (0.5%)	
Other	2,152 (0.7%)	2,101 (0.7%)	51 (0.4%)	
Missing	22,147 (7.2%)	21,742 (7.4%)	405 (3.1%)	
**Body mass index**, N (%)				< 0∙0001^b^
Normal weight	45,149 (14.7%)	43,025 (14.6%)	1,970 (14.9%)	
Obese	43,596 (14.2%)	42,172 (14.3%)	1,466 (11.1%)	
Overweight	38,847 (12.6%)	37,385 (12.7%)	1,422 (10.8%)	
Underweight	4,951 (1.6%)	4,629 (1.6%)	327 (2.5%)	
Missing	174,933 (56.9%)	167,048 (56.8%)	8,032 (60.8%)	
**Charlson Comorbidity Index**, N (%)				< 0∙0001^b^
0	211,182 (68.7%)	202,630 (68.9%)	8,923 (67.5%)	
1–2	29,696 (9.7%)	28,250 (9.6%)	1,919 (14.5%)	
3–4	4,741 (1.5%)	4,597 (1.6%)	202 (1.5%)	
5+	4,409 (1.4%)	4,322 (1.5%)	101 (0.8%)	
Missing	57,448 (18.7%)	54,460 (18.5%)	2,072 (15.7%)	
**Smoking status**, N (%)				< 0∙0001^b^
Current smoker	78,596 (25.6%)	73,615 (25%)	4,972 (37.6%)	
Former smoker	45,999 (15%)	44,778 (15.2%)	1,231 (9.3%)	
Non-smoker	68,602 (22.3%)	66,478 (22.6%)	1,953 (14.8%)	
Missing	114,279 (37.2%)	109,388 (37.2%)	5,061 (38.3%)	

*For comparison of characteristics between MDD with and without moderate-to-high-suicide-intent subgroups

^a^Student’s t-test. ^b^Pearson’s chi-squared test

^†^Percentages computed thanks to the repartition of CPRD active patients in 2013, available at https://academic.oup.com/ije/article/44/3/827/632531

### Incidence of moderate-to-high-suicide-intent

The incidence rate of moderate-to-high-suicide-intent in patients with MDD was 12 per 1,000 person-years in the overall study population, 15 per 1,000 person-years in males and 10 per 1,000 person-years in females. The 18–19 age group accounted for the highest incidence rate with 38 per 1,000 person-years, followed by 22 and 15 per 1,000 person-years for the 20–24 and 25–29 age groups, respectively, decreasing thereafter with age (7 per 1,000 person-years in the 85–89 age group).

### Clinical outcomes

A diagnosis of anxiety disorder was present in 32.4% of the overall population with MDD and was significantly more frequent in the group with moderate-to-high-suicide-intent (44% [2,247/9,562]) than in the group without suicide intent (31.9% [77,331/242,223]) (*p* < 0.0001). The diagnosis of substance use disorder was also more common amongst MDD with moderate-to-high-suicide-intent group (45.7% [4,374/9,562] vs. 19.4% [47,033/242,223]) (*p* < 0.0001).

The overall mortality rate was 2.6% (*n* = 7,343) and was similar between patients with MDD with and without moderate-to-high-suicide-intent (2.5% [307/12,169] vs. 2.6% [7,036/275,006]). Death from suicide was recorded in 0.9% (*n* = 106) of patients with MDD with moderate-to-high-suicide-intent and in none of the patients with MDD without suicide intent, while death from other causes was registered in 1.6% and 2.6% of patients, respectively.

### Healthcare resource utilisation (HCRU)

As shown in Table 2, HCRU during the first follow-up year was higher for patients with MDD with moderate-to-high-suicide-intent as compared with those without (mean GP visits 38.5 vs. 29.4, face-to-face 11.6 vs. 10.3, telephone 1.5 vs. 1.0). Psychiatric visits were recorded in 40.4% of patients with MDD and moderate-to-high-suicide-intent and in only 5.3% of those without suicide intent, with an average of 3.6 vs. 0.3 visits. The mean number of psychiatric outpatient services was 1.5 vs. 0.06 for patients with MDD with and without moderate-to-high-suicide-intent. Moreover, notable differences were also found in the percentage of patients with accident and emergency visits between patients with MDD with and without moderate-to-high-suicide-intent (62.1% vs. 17.9%; mean visits 1.5 vs. 0.3). The need of inpatient care was also higher amongst patients with MDD with moderate-to-high-suicide-intent than amongst those without, both in the percentage of patients with ≥ 1 hospitalisation (86.2% vs. 26.3%) and in the use of inpatient psychiatric services (11.5% vs. 0.4%). Requirements of ICU and Psychiatric ICU admissions were also greater among patients with MDD with moderate-to-high-suicide-intent (3.2% vs. 0.6% and 0.1% vs. 0%, respectively).

**Table 2:** Healthcare resource utilisation (HCRU) in patients with major depressive disorder (MDD) without and with moderate-to-high-suicide-intent

Variables	HCRU during the first year of follow-up					4 weeks after the index date	
	MDD without moderate- to-high suicide intent (*n* = 242,223)		MDD with moderate- to-high suicide intent (*n* = 9,562)		*P*-value for the difference in proportions and/or means^**^	MDD with moderate-to-high-suicide-intent (*n* = 12,721)	
	**N (%)**	**Mean (SD)**	**N (%)**	**Mean (SD)**		**N (%)**	**Mean (SD)**
GP consultations	242,020 (99.9%)	29.4 (20.5)	9,529 (99.7%)	38.5 (25.2)	< 0∙0001, < 0∙0001	12,532 (98.5%)	6.1 (3.1)
Face-to-face [20]		10.3 (8.1)		11.6 (9.9)	< 0∙0001		
Telephone		1.0 (8.1)		1.5 (3.5)	< 0∙0001		
Other (including administrative tasks)		20.7 (17.1)		29.0 (21.2)	< 0∙0001		
IAPT services	250 (0.1%)	0 (0.04)	68 (0.8%)	0.01 (0.1)	< 0∙0001, < 0∙0001	10 (0.1%)	0 (0.04)
Psychiatric outpatient services (CRHT/CMHT)^*^	2,384 (1%)	0.06 (1.2)	1,600 (17.8%)	1.5 (6.6)	< 0∙0001, < 0∙0001	1,880 (14.9%)	0.6 (2.4)
Psychiatric visits^*^	12,117 (5.3%)	0.3 (2.5)	3,633 (40.4%)	3.6 (11.1)	< 0∙0001, < 0∙0001	3,861 (30.5%)	1.0 (2.8)
Accident and emergency visits	43,438 (17.9%)	0.3 (0.9)	5,941 (62.1%)	1.5 (2.8)	< 0∙0001, < 0∙0001	6,514 (51.2%)	0.6 (0.8)
Patients with ≥ 1 hospitalisation	63,649 (26.3%)		8,242 (86.2%)		< 0∙0001	10,413 (81.9%)	
Patients with ≥ 1 psychiatric in-patient services	957 (0.4%)		1,101 (11.5%)		< 0∙0001	1,109 (8.7%)	
Patients with ≥ 1 ICU admission	1,384 (0.6%)		309 (3.2%)		< 0∙0001	334 (2.6%)	
Patients with ≥ 1 Psychiatric ICU admission^*^	5 (0.0%)		5 (0.1%)		< 0∙0001	6 (0%)	
Single Point of Access*	0 (0)%	0 (0)	0 (0)%	0 (0)	***	0 (0%)	0 (0)
24/7 Crisis Response Line*	0 (0)%	0 (0)	0 (0)%	0 (0)	***	0 (0%)	0 (0)
Referral to CMHT	2111 (0.9%)	0.01 (0.1)	231 (2.4%)	0.03 (0.17)	< 0.0001, < 0.0001	127 (1%)	0.01 (0.1)
Referral to improving access to psychological therapies (IAPT)	888 (0.4%)	0 (0.07)	32 (0.3%)	0 (0.06)	0.6116, 0.5949	14 (0.1%)	0 (0.03)
Referral to Single Point of Access	2 (0%)	0 (0)	0 (0%)	0 (0)	***, 0.1573	0 (0%)	0 (0)

SD: standard deviation; GP: general practitioner; IAPT: Improving Access to Psychological Therapies; CRHT: Crisis Resolution Home Treatment Team; CMHT: Community Mental Health Team; ICU; intensive care unit

^*^1-year of follow-up before the end of the study period (30th November 2015) *n* = 230,039 MDD without moderate-to-high-suicide-intent, *n* = 8,988 MDD with moderate-to-high-suicide-intent; within 4-weeks after the index date, *n* = 12,645 MDD with moderate-to-high-suicide-intent

^**^P-value of Pearson’s chi-squared test for proportions and of Student’s t-test for means

^***^ P-value not computed because of insufficient data

In patients with MDD and moderate-to-high-suicide-intent, the analysis of HCRU within four weeks after the index diagnosis showed GP consultations in 98.5% of patients (mean 6.1 visits), 30.5% required psychiatric visits, 81.9% had ≥ 1 hospitalisation for any reason, and 8.7% used psychiatric inpatient services. Also, 2.6% required ≥ 1 ICU admission (Table 2).

### Treatment patterns

In the 12,695 patients with MDD and moderate-to-high-suicide-intent, 7,154 (56.4%) had either a prescription of an antidepressant medication within 30 days of the index date (citalopram 34%, fluoxetine 20%, mirtazapine 15%, and sertraline 14%), or in combination with antipsychotics (*n* = 355, 2.8%), anticonvulsants (*n* = 89, 0.7%), and lithium (*n* = 17, 0.1%). As shown in Table 3, citalopram was the most frequently prescribed antidepressant (index pharmacological treatment [i.e., prescribed within 30 days of the index date] 19.2%, subsequent treatment 7.6%). Amongst patients initiating an index drug treatment, 54.8% (*n* = 3,918) switched treatment, with a 25% probability of switching within three months after the index date. Only 14 (0.1%) patients received ECT. Non-pharmacological/non-interventional treatments during the first year of follow-up included psychotherapy in 84 patients (0∙7%), occupational therapy in 22 (0∙2%), and cognitive behavioural therapy in 15 (0∙1%).

**Table 3:** Antidepressants use among patients with MDD and moderate-to-severe-suicide-intent and minimum 30 days of follow-up (*n* = 12,695)

Antidepressant agents	Index treatment	Subsequent treatment
Selective serotonin reuptake inhibitors (SSRIs)		
Citalopram	2,434 (19.2%)	971 (7.6%)
Fluoxetine	1,463 (11.5%)	527 (4.2%)
Sertraline	980 (7.7%(	738 (5.8%)
Escitalopram	195 (1.5%)	110 (0.9%)
Paroxetine	189 (1.5%)	73 (0.6%)
Fluxovamine	1 (0%)	5 (0%)
**Tricyclic antidepressants**		
Amitryptiline	43 (0.3%)	27 (0.2%)
Dosulepin	56 (0.4%)	44 (0.3%)
Lofepramine	24 (0.2%)	26 (0.2%)
Nortryptiline	16 (0.1%)	26 (0.2%)
Clomipramine	19 (0.1%)	19 (0.1%)
Imipramine	3 (0%)	7 (0.1%)
Doxepin	3 (0%)	5 (0%)
Trimipramine	4 (0%)	6 (0%)
Amoxapine	0	0
Butryptiline	0	0
Desipramine	0	0
Iprindole	0	0
Protriptyline	0	0
Viloxacine	0	0
**Selective norepinephrine reuptake inhibitors (SNRIs)**		
Venlafaxine	561 (4.4%)	493 (3.9%)
Duloxetine	157 (1.2%)	142 (1.1%)
**Tetracyclic antidepressants**		
Mirtazapine	1,098 (8.6%)	881 (6.9%)
Trazodone	127 (1%)	141 (1.1%)
Mianserin	1 (0%)	1 (0%)
Maprotiline	0	0
**Monoamino oxidase inhibitors (MAOIs)**		
Moclobemide	4 (0%)	3 (0%)
Phenelzine	1 (0%)	0
Iproniazid	0	0
Isocarboxazid	0	0
Tranylcypromine	0	0
**Other antidepressants**		
Agomelatine	2 (0%)	1 (0%)
Reboxetine	4 (0%)	4 (0%)
Nefazodone	0	0
Vortioxetine	0	1 (0%)

## Discussion

### Key findings

Through this study we were able to compare the clinical profile and the HCRU of patients with MDD with and without moderate-to-high-suicide-intent in England. To our knowledge, this is one of the largest cohorts of patients with MDD with moderate-to-high-suicide-intent and the first study evaluating their burden in England. In this study using CPRD-HES-ONS-MHSDS data between April 2007 and November 2015, moderate-to-high-suicide-intent occurred in 4.3% of patients with MDD. Estimates of the prevalence of imminent risk of suicide are scarce, and a previous study based on a global online community resource (“PatientsLikeMe”) found that amongst 12,229 users, 266 (2.3%) reported symptoms related to suicidality [18]. Although suicidal ideation is concerning and common, many people who experience this have no intention to act on their suicidal thoughts. The patients who have formed an intention to act on their suicidal thoughts are a smaller, more vulnerable, and higher risk group of patients. In the 2017 National Health and Wellness Survey in five European countries, suicidal ideation was reported by 27∙3% of the 3,308 individuals diagnosed with MDD [19]. In this survey, however, MDD diagnoses were based on patient-reported data, and patients with suicidal thoughts were evaluated, whereas our study focused on patients with moderate-to-high-suicide-intent.

Patients with MDD and moderate-to-high-suicide-intent were younger than those without (39 vs. 44.8 years) and although most patients were female, the proportion of males was greater among those with moderate-to-high-suicide-intent. In a previous study in England of 41,375 patients with MDD to assess treatment-resistant depression, the mean age was 44 years and 32% were male [16], which is similar to our average age of 44.8 years and 35.2% of males. Patients with MDD and moderate-to-high-suicide-intent were also more commonly diagnosed with substance abuse (46% vs. 19%) and anxiety (44% vs. 32%) at follow-up, reflecting another contributing factor to the burden of MDD disease. In the study of users of the global web platform “PatientsLikeMe”, anxiety disorder was reported by 63.1% of patients with MDD with suicidal ideation (vs. 44% in our patients with MDD with moderate-to-high-suicide-intent) and 44.3% in the MDD cohort (vs. 32% in our patients with MDD without suicide intent). The higher percentage of anxiety disorder in the “PatientsLikeMe” study may be explained by the difference between suicide intent and suicidal ideation and data based on patient reporting, in contrast to our series based on clinical diagnostic codes.

This study clearly shows that patients with MDD and moderate-to-high-suicide-intent incurred greater HCRU compared to those without. During the first year of follow-up, the average number of GP consultations, overall (38.5 vs. 29.4), face-to-face visits (11.6 vs. 10.3), telephone visits (1.5 vs. 1.0) and others (29 vs. 20.7) was higher. These findings are similar to data reported in a study using the CPRD database from 2000 to 2012, where patients with severe mental illness had an annual average of 10.9 face-to-face visits, 1.2 telephone consultations, and 37.3 other consultations [20]. Moreover, within four weeks after the index date, we found that patients with MDD with moderate-to-high-suicide-intent visited their GP on average 6.1 times.

Regarding psychiatric outpatient services, only 17.8% of patients with MDD and moderate-to-high-suicide-intent used CRHT/CMHT. These findings may be due to underreporting, use of paper records and patients encountering these services outside of the network of practices contributing to MHSDS data but may also indicate use of other services that are not currently captured in the study datasets. Capturing the data accurately is a key issue, indeed low numbers of patients were found to be using “Single point of access” and “24/7 Crisis Response Line”, potentially due to these codes being used only when the team’s primary function is the mentioned service. It is likely that in most cases, those services are not the primary function of the team, and thus their specific use cannot be adequately measured.

In the year following their index date, a high proportion of patients with MDD and moderate-to-high-suicide-intent required at least one hospital admission (81.9%), whereas the proportion was 26.3% for patients with MDD without suicide intent. Admission to the Psychiatric ICU and inpatient psychiatric services, however, were less frequent (0.1% and 11.5%, respectively), but still more frequent than for patients without suicide intent (0.0% and 0.4%, respectively). In a systematic review, typical psychosis in Psychiatric ICU patients was due to schizophrenia or mania [21]. In the cross-sectional survey in five European countries, the group of patients with suicidal ideation showed a higher HCRU with statistically significant differences compared to both the non-suicidal ideation group and the general population [19].

Citalopram, fluoxetine, mirtazapine, and sertraline were the most prescribed index and subsequent pharmacological treatments amongst patients with MDD and moderate-to-high-suicide-intent. These findings are consistent with previous studies evaluating antidepressant use amongst patients with MDD in England [16, 22]. The use of non-pharmacological/non-interventional treatments was very low, which may reflect a limitation of the datasets used to accurately capture these interventions, that may have been administered in centres outside these datasets or under-recorded in the patients’ medical records. In a retrospective analysis of patients with MDD and treatment-resistant depression in the same linked datasets, the recorded use of non-pharmacological/non-interventional treatments was also very limited [16].

### Strengths and limitations

Limitations of the study are inherent to the nature of the databases and of real-world evidence generally, such as the use of Read and ICD-10 codes to identify patients due to the complexity of identifying individuals at imminent risk of suicide, potential miscoding, and multiple situations that can be covered by one diagnosis. However, codes and levels of suicide intent were validated by clinical experts. It is possible that a patient’s MDD diagnosis would have been recorded by a physician as a symptom without using a diagnostic code and therefore, not indexed in the study. Some patients (private patients, prisoners, some residential homes, and homeless) are not represented in the CPRD. The datasets might not capture all HCRU and, in this respect, particularly CMHT and CRHT use appears to be underreported, as well as the use of non-pharmacological/non-interventional treatments. The use of CMHT and CRHT should be considered for future research given patients with MDD and moderate-to-high suicide-intent are under care of these services. Certain baseline results are inconclusive due to many missing values for several characteristics, such as BMI and smoking status. Our study described significant differences in HCRU between patients with MDD with or without moderate-to-high-suicide-intent, and further studies using more comprehensive data would enable to analyse the impact of moderate-to-high-suicide-intent on the clinical and economic burden. Treatment patterns were evaluated by prescriptions written by GPs, which did not capture whether the prescription was dispensed or consumed. Pharmacological treatments prescribed in secondary care settings were also not captured. It was also not possible to deduce the line of therapy of the index and subsequent treatment, as access to patients’ full medical history was not feasible. Data on the clinical status (response to treatment, remission, etc.) was also not available in the database.

Study strengths include the value of linked datasets from four national sources that allowed the assessment of a large sample of patients with MDD with and without moderate-to-high-suicide-intent, reflecting real-world care patterns in a nationally representative population of England. The exclusion of patients with other mental illness (psychosis, schizophrenia, mania, bipolar disorder, or dementia) resulted in a study sample that could be used specifically to assess the burden of moderate-to-high-suicide patients with MDD. These features support the clinical relevance of the study findings.

## Conclusions

In conclusion, the presence of moderate-to-high-suicide-intent in patients with MDD was associated with a younger age, higher proportion of males, higher probability of suffering from concurrent anxiety and substance abuse disorders, and notably higher HCRU as compared to patients with MDD without suicide intent. Moreover, more than half of patients with MDD and moderate-to-high-suicide-intent switched treatment, many of them within three months of the initial antidepressant prescription. This could illustrate the need for more effective treatments or a more personalised approach to treatment, to ensure patients receive the best-suited therapy in first line. These results suggest a greater need for effective medical care and appropriate treatments for patients with moderate-to-high-suicide-intent, which could help reduce associated symptoms, mortality due to suicide, and HCRU.

### Electronic supplementary material

Below is the link to the electronic supplementary material.

Supplementary Material 1

## Data Availability

The linked CPRD-HES-ONS-MHSDS data was provided by CPRD. Due to data licensing agreements, the data are not available to include in the submission.

## Abbreviations

BMI: Body Mass Index
CCI: Charlson Comorbidity Index
CMHT: Community Mental Health Team
CPG: Clinical Practice Guidelines
CPRD: Clinical Practice Research Datalink
CRHT: Crisis Resolution Home Treatment Team
DALY: Disability Adjusted Life Years
DBS: Deep Brain Stimulation
ECT: Electroconvulsive Therapy
GP: General Practitioner
HCRU: Healthcare Resource Utilisation
HES: Hospital Episode Statistics
IAPT: Improving Access to Psychological Therapies
ICD-10: International Classification of Disease-10th revision
ICU: Intensive Care Unit
MDD: Major Depressive Disorder
MHSDS: Mental Health Services Data Set
NCISH: National Confidential Inquiry into Suicide and Safety in Mental Health
ONS: Office for National Statistics
TMS: Transcranial Magnetic Stimulation
UK: United Kingdom
VNS: Vagus Nerve Stimulation
