# Supplementary material for: Impact of moderate-to-high-suicide-intent in major depressive disorder: a retrospective cohort study on patient characteristics and healthcare resource utilisation in England
Source: BMC Psychiatry. 2024 Aug 23;24:576. doi: 10.1186/s12888-024-05961-3 (PMC11342576; doi:10.1186/s12888-024-05961-3)
Supplement: Supplementary file 1 — Supplementary Material 1 [file 12888_2024_5961_MOESM1_ESM.docx]

**Supplementary material**

**Table S1: Populations considered in the different analyses**

| **Analysis** | **Condition** | **MDD without moderate-to-high-suicide-intent**  **(n=294,259)** | **MDD with moderate-to-high-suicide-intent**  **(n=13,217)** |
| --- | --- | --- | --- |
| HCRU and non-pharmacological and/or non-interventional therapies | 1 year of follow-up | 242,223 | 9,562 |
| HCRU and non-pharmacological and/or non-interventional therapies for outcomes using MHSDS data | 1 year of follow-up before 30^th^ November 2015 | 230,039 | 8,988 |
| HCRU in the first four weeks following index date (initial diagnosis of moderate-to-high-suicide-intent) | 4 weeks of follow-up | Not applicable | 12,721 |
| HCRU in the first four weeks following index date (initial diagnosis of moderate-to-high-suicide-intent) using MHSDS data | 4 weeks of follow-up before 30^th^ November 2015 | Not applicable | 12,645 |
| Pharmacological treatment | 30 days of follow-up | Not applicable | 12,695 |
| Mortality | Index date at least 1 year before the end of the study period (30^th^ November 2015) | 275,006 | 12,169 |

MDD: major depressive disorder; HCRU: healthcare resource utilisation; MHSDS: Mental Health Services Data Set.

**Table S2: National Health Service (NHS) digital eight-step linkage algorithm**

| **Step** | **NHS number** | **Date of birth** | **Sex** | **Postcode** |
| --- | --- | --- | --- | --- |
| 1 | Exact | Exact | Exact | Exact |
| 2 | Exact | Exact | Exact |  |
| 3 | Exact | Partial | Exact | Exact |
| 4 | Exact | Partial | Exact |  |
| 5 | Exact |  |  |  |
| 6^*^ |  | Exact |  | Exact |
| 7^†^ |  | Exact | Exact | Exact |
| 8 | Exact |  | Exact | Exact |

^*^where NHS number does not contradict the match, data of birth is not 1st of January and postcode not on the communal establishment list; ^†^where NHS number does not contradict the match and data of birth is not 1st of January.

**Table S3: Read and International Classification of Diseases-10th revision (ICD-10) codes for the diagnosis of major depressive disorder (MDD)**

| **Coding System** | **Code** | **Description** |
| --- | --- | --- |
| Read | 1465,00 | H/O: depression |
| Read | 2257,00 | O/E - depressed |
| Read | 1B17.00 | Depressed |
| Read | 1B17.11 | C/O - feeling depressed |
| Read | 1B1U.00 | Symptoms of depression |
| Read | 1B1U.11 | Depressive symptoms |
| Read | 1BT..00 | Depressed mood |
| Read | 1JJ..00 | Suspected depression |
| Read | 8HHq.00 | Referral for guided self-help for depression |
| Read | E112.00 | Single major depressive episode |
| Read | E112.11 | Agitated depression |
| Read | E112.12 | Endogenous depression first episode |
| Read | E112.13 | Endogenous depression first episode |
| Read | E112.14 | Endogenous depression |
| Read | E112000 | Single major depressive episode, unspecified |
| Read | E112200 | Single major depressive episode, moderate |
| Read | E112300 | Single major depressive episode, severe, without psychosis |
| Read | E112z00 | Single major depressive episode NOS |
| Read | E113.00 | Recurrent major depressive episode |
| Read | E113.11 | Endogenous depression - recurrent |
| Read | E113000 | Recurrent major depressive episodes, unspecified |
| Read | E113200 | Recurrent major depressive episodes, moderate |
| Read | E113300 | Recurrent major depressive episodes, severe, no psychosis |
| Read | E113700 | Recurrent depression |
| Read | E113z00 | Recurrent major depressive episode NOS |
| Read | E11y200 | Atypical depressive disorder |
| Read | E11z200 | Masked depression |
| Read | E135.00 | Agitated depression |
| Read | E204.00 | Neurotic depression reactive type |
| Read | E2B..00 | Depressive disorder NEC |
| Read | E2B1.00 | Chronic depression |
| Read | Eu32.00 | [X]Depressive episode |
| Read | Eu32.11 | [X]Single episode of depressive reaction |
| Read | Eu32.12 | [X]Single episode of psychogenic depression |
| Read | Eu32.13 | [X]Single episode of reactive depression |
| Read | Eu32100 | [X]Moderate depressive episode |
| Read | Eu32200 | [X]Severe depressive episode without psychotic symptoms |
| Read | Eu32211 | [X]Single episode agitated depressn w'out psychotic symptoms |
| Read | Eu32212 | [X]Single episode major depression w'out psychotic symptoms |
| Read | Eu32213 | [X]Single episode vital depression w'out psychotic symptoms |
| Read | Eu32600 | [X]Major depression, moderately severe |
| Read | Eu32700 | [X]Major depression, severe without psychotic symptoms |
| Read | Eu32y00 | [X]Other depressive episodes |
| Read | Eu32y11 | [X]Atypical depression |
| Read | Eu32y12 | [X]Single episode of masked depression NOS |
| Read | Eu32z00 | [X]Depressive episode, unspecified |
| Read | Eu32z11 | [X]Depression NOS |
| Read | Eu32z12 | [X]Depressive disorder NOS |
| Read | Eu32z13 | [X]Prolonged single episode of reactive depression |
| Read | Eu32z14 | [X] Reactive depression NOS |
| Read | Eu33.00 | [X]Recurrent depressive disorder |
| Read | Eu33.11 | [X]Recurrent episodes of depressive reaction |
| Read | Eu33.12 | [X]Recurrent episodes of psychogenic depression |
| Read | Eu33.13 | [X]Recurrent episodes of reactive depression |
| Read | Eu33100 | [X]Recurrent depressive disorder, current episode moderate |
| Read | Eu33200 | [X]Recurr depress disorder cur epi severe without psyc sympt |
| Read | Eu33211 | [X]Endogenous depression without psychotic symptoms |
| Read | Eu33212 | [X]Major depression, recurrent without psychotic symptoms |
| Read | Eu33214 | [X]Vital depression, recurrent without psychotic symptoms |
| Read | Eu33y00 | [X]Other recurrent depressive disorders |
| Read | Eu33z00 | [X]Recurrent depressive disorder, unspecified |
| Read | Eu33z11 | [X]Monopolar depression NOS |
| Read | Eu34111 | [X]Depressive neurosis |
| Read | Eu34113 | [X]Neurotic depression |
| ICD-10 | F32.1 | Moderate depressive episode |
| ICD-10 | F32.2 | Severe depressive episode without psychotic symptoms |
| ICD-10 | F32.8 | Other depressive episodes |
| ICD-10 | F32.9 | Depressive episode, unspecified |
| ICD-10 | F33.1 | Recurrent depressive disorder, current episode moderate |
| ICD-10 | F33.2 | Recurrent depressive disorder, current episode severe without psychotic symptoms |
| ICD-10 | F33.8 | Other recurrent depressive disorders |
| ICD-10 | F33.9 | Recurrent depressive disorder, unspecified |

**Table S4: Read codes of suicide by level of intent**

| **Read code** | **Read term** | **Level of intent** |
| --- | --- | --- |
| 14K1.00 | Intentional overdose of prescription only medication | High |
| 1B19.00 | Suicidal | Undefinable |
| 1B19.11 | Suicidal - symptom | Undefinable |
| TK3z.00 | Suicide + selfinflicted inj by hang/strangle/suffocate NOS | No event |
| TK3y.00 | Suicide + selfinflicted inj oth mean hang/strangle/suffocate | No event |
| TKx0z00 | Suicide + selfinflicted inj-jump/lie before moving obj NOS | No event |
| 1BD1.00 | Suicidal ideation | Undefinable |
| TK31.00 | Suicide + selfinflicted injury by suffocation by plastic bag | No event |
| TKx0.00 | Suicide + selfinflicted injury-jump/lie before moving object | No event |
| TKx0000 | Suicide + selfinflicted injury-jumping before moving object | No event |
| TK06.00 | Suicide + selfinflicted poisoning by agricultural chemical | No event |
| 1BD3.00 | Suicidal plans | High |
| TK01.00 | Suicide + selfinflicted poisoning by barbiturates | No event |
| TK07.00 | Suicide + selfinflicted poisoning by corrosive/caustic subst | No event |
| TK1z.00 | Suicide + selfinflicted poisoning by domestic gases NOS | No event |
| 1BD4.00 | Suicide risk | Moderate |
| TK10.00 | Suicide + selfinflicted poisoning by gas via pipeline | No event |
| TK2z.00 | Suicide + selfinflicted poisoning by gases and vapours NOS | No event |
| TK1..00 | Suicide + selfinflicted poisoning by gases in domestic use | No event |
| TK11.00 | Suicide + selfinflicted poisoning by liquified petrol gas | No event |
| 1BD5.00 | High suicide risk | High |
| 1BD6.00 | Moderate suicide risk | Moderate |
| 1BDC.00 | Intent of deliberate self harm with detailed plans | High |
| TK2y.00 | Suicide + selfinflicted poisoning by other gases and vapours | No event |
| TK2..00 | Suicide + selfinflicted poisoning by other gases and vapours | No event |
| 1JP..00 | Suspected drug overdose | Undefinable |
| SL...14 | Overdose of biological substance | High |
| SL...15 | Overdose of drug | Undefinable |
| TK...00 | Suicide and selfinflicted injury | High |
| TK01000 | Suicide and self inflicted injury by Amylobarbitone | No event |
| TK01100 | Suicide and self inflicted injury by Barbitone | No event |
| TK01z00 | Suicide and self inflicted injury by barbiturates | No event |
| TK01400 | Suicide and self inflicted injury by Phenobarbitone | No event |
| TK...11 | Cause of overdose - deliberate | High |
| TK...13 | Poisoning - self-inflicted | High |
| TKx5.00 | Suicide and selfinflicted injury by crashing motor vehicle | No event |
| TKx6.00 | Suicide and selfinflicted injury by crashing of aircraft | No event |
| TK...14 | Suicide and self harm | High |
| TK...15 | Attempted suicide | High |
| TK0..00 | Suicide + selfinflicted poisoning by solid/liquid substances | High |
| TK00.00 | Suicide + selfinflicted poisoning by analgesic/antipyretic | High |
| TKx4.00 | Suicide and selfinflicted injury by electrocution | No event |
| TKx3.00 | Suicide and selfinflicted injury by extremes of cold | No event |
| TK5..00 | Suicide and selfinflicted injury by firearms and explosives | No event |
| TK5z.00 | Suicide and selfinflicted injury by firearms/explosives NOS | No event |
| TK02.00 | Suicide + selfinflicted poisoning by oth sedatives/hypnotics | High |
| TK52.00 | Suicide and selfinflicted injury by hunting rifle | No event |
| TK03.00 | Suicide + selfinflicted poisoning tranquilliser/psychotropic | High |
| TK53.00 | Suicide and selfinflicted injury by military firearms | No event |
| TK54.00 | Suicide and selfinflicted injury by other firearm | No event |
| TKx..00 | Suicide and selfinflicted injury by other means | No event |
| TK04.00 | Suicide + selfinflicted poisoning by other drugs/medicines | High |
| TKxy.00 | Suicide and selfinflicted injury by other specified means | No event |
| TKx2.00 | Suicide and selfinflicted injury by scald | No event |
| TK51.00 | Suicide and selfinflicted injury by shotgun | No event |
| TK05.00 | Suicide + selfinflicted poisoning by drug or medicine NOS | High |
| TKx7.00 | Suicide and selfinflicted injury caustic subst, excl poison | No event |
| TK0z.00 | Suicide + selfinflicted poisoning by solid/liquid subst NOS | High |
| TK21.00 | Suicide and selfinflicted poisoning by other carbon monoxide | No event |
| TK1y.00 | Suicide and selfinflicted poisoning by other utility gas | No event |
| TK20.00 | Suicide + selfinflicted poisoning by motor veh exhaust gas | High |
| TK72.00 | Suicide+selfinflicted injury-jump from natural sites | No event |
| TK71.00 | Suicide+selfinflicted injury-jump from oth manmade structure | No event |
| TK70.00 | Suicide+selfinflicted injury-jump from residential premises | No event |
| ZX1Q.00 | Throwing self in front of train | No event |
| TK3..00 | Suicide + selfinflicted injury by hang/strangulate/suffocate | High |
| U2B6.00 | [X]Int self harm by jump from high place indust/constr area | No event |
| U2By.00 | [X]Int self harm by jump from high place occ oth specif plce | No event |
| U2Bz.00 | [X]Int self harm by jump from high place occ unspecif place | No event |
| U282.00 | [X]Int self harm by steam hot vapor/obj sch/ins/pub adm area | No event |
| U2Cy.00 | [X]Int self harm jump/lying bef mov obje occ oth specif plce | No event |
| U2C1.00 | [X]Int self harm jump/lying befr mov obje occ resid instit'n | No event |
| U2C4.00 | [X]Int self harm jump/lying befr mov obje occ street/highway | No event |
| U241.00 | [X]Int self harm rifl s'gun/lrg frarm disch occ resid instit | No event |
| U200600 | [X]Int self pois nonopioid analgesic indust/construct area | No event |
| U20Az00 | [X]Int self pois org solv,halogen hydrocarb, unspec place | No event |
| U20Ay00 | [X]Int self pois org solv,halogen hydrocarb,oth spec place | No event |
| U209y00 | [X]Int self poison alcohol other spec place | No event |
| U205y00 | [X]Int self poison narcotic drug other spec place | No event |
| U200y00 | [X]Int self poison nonopioid analgesic other spec place | No event |
| U20A400 | [X]Int self poison org solvent,halogen hydrocarb,in highway | No event |
| U208y00 | [X]Int self poison oth/unsp drug/medic other spec place | No event |
| U20By00 | [X]Int self poison other gas/vapour other spec place | No event |
| U20B200 | [X]Int self poison other gas/vapour school/pub admin area | No event |
| U20Cy00 | [X]Int self poison pesticide other spec place | No event |
| U204y00 | [X]Int self poison psychotropic drug other spec place | No event |
| U202y00 | [X]Int self poison sedative hypnotic other spec place | No event |
| U20y200 | [X]Int self poison unspecif chemical school/pub admin area | No event |
| U209000 | [X]Int self poison/exposure to alcohol at home | No event |
| U201000 | [X]Int self poison/exposure to antiepileptic at home | No event |
| U206000 | [X]Int self poison/exposure to hallucinogen at home | No event |
| TK30.00 | Suicide and selfinflicted injury by hanging | High |
| U200000 | [X]Int self poison/exposure to nonopioid analgesic at home | No event |
| U207000 | [X]Int self poison/exposure to oth autonomic drug at home | No event |
| U208000 | [X]Int self poison/exposure to oth/unsp drug/medicam home | No event |
| U20B000 | [X]Int self poison/exposure to other gas/vapour at home | No event |
| TK4..00 | Suicide and selfinflicted injury by drowning | High |
| U20C000 | [X]Int self poison/exposure to pesticide at home | No event |
| TK6..00 | Suicide and selfinflicted injury by cutting and stabbing | High |
| U202000 | [X]Int self poison/exposure to sedative hypnotic at home | No event |
| U20y000 | [X]Int self poison/exposure to unspecif chemical at home | No event |
| U242.00 | [X]Int slf hrm rifl s'gun/lrg frarm dis sch/ins/pub adm area | No event |
| U212.00 | [X]Inten slf harm hang strang/suffc sch oth ins/pub adm area | No event |
| U2A2.00 | [X]Intent self harm blunt obj occ sch oth ins/pub adm area | No event |
| U2Dz.00 | [X]Intent self harm by crash motor vehic occ unspecif place | No event |
| U2D4.00 | [X]Intent self harm by crash motor vehicl occ street/highway | No event |
| U2D0.00 | [X]Intent self harm by crash of motor vehicl occurrn at home | No event |
| U22y.00 | [X]Intent self harm by drown/submersn occ oth specif place | No event |
| U22z.00 | [X]Intent self harm by drown/submersn occ unspecified place | No event |
| U220.00 | [X]Intent self harm by drowning/submersion occurrn at home | No event |
| U221.00 | [X]Intent self harm by drowning/submersn occ resid instit'n | No event |
| U216.00 | [X]Intent self harm by hang strangl/suffc indust/constr area | No event |
| U213.00 | [X]Intent self harm by hang strangl/suffc sport/athlet area | No event |
| U210.00 | [X]Intent self harm by hanging strangulat/suffocat occ home | No event |
| TK60.00 | Suicide and selfinflicted injury by cutting | High |
| TK60100 | Self inflicted lacerations to wrist | Undefinable |
| TK60111 | Slashed wrists self inflicted | Undefinable |
| U211.00 | [X]Intent self harm by hangng strangult/suffoct resid instit | No event |
| U2B1.00 | [X]Intent self harm by jump from high place occ resid instit | No event |
| TK61.00 | Suicide and selfinflicted injury by stabbing | High |
| TK6z.00 | Suicide and selfinflicted injury by cutting and stabbing NOS | High |
| U2B0.00 | [X]Intent self harm by jumping from high place occ at home | No event |
| U25..00 | [X]Intent self harm by other/unspecified firearm discharge | No event |
| U24..00 | [X]Intent self harm by rifle shotgun/larger firearm disch | No event |
| U296.00 | [X]Intent self harm by sharp object occ indust/constr area | No event |
| U295.00 | [X]Intent self harm by sharp object occ trade/service area | No event |
| U27y.00 | [X]Intent self harm by smoke fire/flame occ oth specif plce | No event |
| U274.00 | [X]Intent self harm by smoke fire/flame occ street/highway | No event |
| U27z.00 | [X]Intent self harm by smoke fire/flames occ unspecif place | No event |
| U2D6.00 | [X]Intent self harm crash motor vehic occ indust/constr area | No event |
| U250.00 | [X]Intent self harm oth/unspecif firearm disch occ at home | No event |
| U292.00 | [X]Intent self harm sharp obj occ sch oth ins/pub adm area | No event |
| U209400 | [X]Intent self pois alcohol in street/highway | No event |
| U206400 | [X]Intent self pois hallucinogen in street/highway | No event |
| U200400 | [X]Intent self pois nonopioid analgesic in street/highway | No event |
| U200500 | [X]Intent self pois nonopioid analgesic trade/service area | No event |
| U20A000 | [X]Intent self pois organ solvent,halogen hydrocarb, home | No event |
| U208400 | [X]Intent self pois oth/unsp drug/medic in street/highway | No event |
| U202400 | [X]Intent self pois sedative hypnotic in street/highway | No event |
| U209z00 | [X]Intent self poison alcohol unspecif place | No event |
| U201z00 | [X]Intent self poison antiepileptic unspecif place | No event |
| U206z00 | [X]Intent self poison hallucinogen unspecif place | No event |
| U205z00 | [X]Intent self poison narcotic drug unspecif place | No event |
| U200100 | [X]Intent self poison nonopioid analgesic at res institut | No event |
| TK7..00 | Suicide and selfinflicted injury by jumping from high place | High |
| U207z00 | [X]Intent self poison oth autonomic drug unspecif place | No event |
| U208z00 | [X]Intent self poison oth/unsp drug/medic unspecif place | No event |
| U20Bz00 | [X]Intent self poison other gas/vapour unspecif place | No event |
| U204100 | [X]Intent self poison psychotropic drug at res institut | No event |
| U204z00 | [X]Intent self poison psychotropic drug unspecif place | No event |
| U202z00 | [X]Intent self poison sedative hypnotic unspecif place | No event |
| U20yz00 | [X]Intent self poison unspecif chemical unspecif place | No event |
| TK7z.00 | Suicide+selfinflicted injury-jump from high place NOS | High |
| U201.00 | [X]Intent self poison/exposure to antiepileptic | No event |
| U206.00 | [X]Intent self poison/exposure to hallucinogen | No event |
| U205.00 | [X]Intent self poison/exposure to narcotic drug | No event |
| TKx1.00 | Suicide and selfinflicted injury by burns or fire | High |
| U207.00 | [X]Intent self poison/exposure to oth autonomic drug | No event |
| U20B.00 | [X]Intent self poison/exposure to other gas/vapour | No event |
| U20C.00 | [X]Intent self poison/exposure to pesticide | No event |
| TKxz.00 | Suicide and selfinflicted injury by other means NOS | High |
| U202.00 | [X]Intent self poison/exposure to sedative hypnotic | No event |
| U20y.00 | [X]Intent self poison/exposure to unspecif chemical | No event |
| U270.00 | [X]Intention self harm by smoke fire/flames occurrn at home | No event |
| U2Az.00 | [X]Intentional self harm by blunt object occ unspecif place | No event |
| U2D..00 | [X]Intentional self harm by crashing of motor vehicle | No event |
| U22..00 | [X]Intentional self harm by drowning and submersion | No event |
| U26..00 | [X]Intentional self harm by explosive material | No event |
| U2B..00 | [X]Intentional self harm by jumping from a high place | No event |
| TKz..00 | Suicide and selfinflicted injury NOS | High |
| U2...13 | [X]Suicide | High |
| U28..00 | [X]Intentional self harm by steam hot vapours / hot objects | No event |
| U20A.00 | [X]Intentional self poison organ solvent,halogen hydrocarb | No event |
| U2...14 | [X]Attempted suicide | High |
| U20A.11 | [X]Self poisoning from glue solvent | No event |
| U20C.12 | [X]Self poisoning with paraquat | No event |
| U20C.11 | [X]Self poisoning with weedkiller | No event |
| U20..00 | [X]Intentional self poisoning/exposure to noxious substances | Undefinable |
| ZX15.00 | Drowning self | No event |
| U20..11 | [X]Deliberate drug overdose / other poisoning | High |
| U200.00 | [X]Intent self poison/exposure to nonopioid analgesic | High |
| U200.11 | [X]Overdose - paracetamol | High |
| U200.12 | [X]Overdose - ibuprofen | Undefinable |
| ZX1R.11 | Jumping in front of vehicle | No event |
| ZX1Q.11 | Jumping under train | No event |
| U200.13 | [X]Overdose - aspirin | Undefinable |
| U200z00 | [X]Intent self poison nonopioid analgesic unspecif place | High |
| ZX1H.00 | Self-asphyxiation | No event |
| U202.11 | [X]Overdose - sleeping tabs | Undefinable |
| ZX1K.00 | Self-incineration | No event |
| ZX1H100 | Self-strangulation | No event |
| ZX1H200 | Self-suffocation | No event |
| ZX1K.11 | Setting fire to self | No event |
| ZX1K.12 | Setting self alight | No event |
| ZX1M.00 | Shooting self | No event |
| U202.12 | [X]Overdose - diazepam | Undefinable |
| U202.13 | [X]Overdose - temazepam | Undefinable |
| U204.00 | [X]Intent self poison/exposure to psychotropic drug | High |
| U204.11 | [X]Overdose - antidepressant | Undefinable |
| U204.12 | [X]Overdose - amitriptyline | Undefinable |
| U204.13 | [X]Overdose - SSRI | Undefinable |
| U204000 | [X]Int self poison/exposure to psychotropic drug at home | High |
| U205000 | [X]Int self poison/exposure to narcotic drug at home | High |
| U202.18 | [X]Overdose - amobarbital | No event |
| U208.00 | [X]Int self poison/exposure to other/unspec drug/medicament | Undefinable |
| U209.00 | [X]Intent self poison/exposure to alcohol | Undefinable |
| U202.17 | [X]Overdose - barbiturate | No event |
| U202.16 | [X]Overdose - benzodiazepine | No event |
| U21..00 | [X]Intent self harm by hanging strangulation / suffocation | High |
| U202.14 | [X]Overdose - flurazepam | No event |
| U21y.00 | [X]Intent self harm by hangng strangul/suffoct oth spec plce | High |
| U202.15 | [X]Overdose - nitrazepam | No event |
| U21z.00 | [X]Intent self harm by hangng strangul/suffoct unspecif plce | High |
| U27..00 | [X]Intentional self harm by smoke, fire and flames | High |
| U290.00 | [X]Intentional self harm by sharp object occurrence at home | Undefinable |
| U2B4.00 | [X]Intent self harm by jump from high place occ street/h'way | High |
| ZX1B200 | Jumping from bridge | No event |
| U2C..00 | [X]Intent self harm by jumping / lying before moving object | High |
| ZX1B300 | Jumping from cliff | No event |
| ZX1B.00 | Jumping from height | No event |
| U41..00 | [X]Hanging strangulation + suffocation undetermined intent | Undefinable |
| ZX18.00 | Hanging self | High |
| ZX1B100 | Jumping from building | High |
| ZX1J.00 | Self-electrocution | High |
| 1BDE.00 | Suicide risk increased from previous level | No event |
| U410.00 | [X]Hanging strangulat+suffocat undet intent occurrn at home | No event |
| ZX1N.00 | Stabbing self | Undefinable |
| 8G6..00 | Anti-suicide psychotherapy | No event |
| 8G6Z.00 | Anti-suicide psychotherapy NOS | No event |
| 9j2..00 | Initiation of suicide risk management document | No event |
| TN80000 | Injury ?accidental, by jumping before moving object | No event |
| TN80.00 | Injury ?accidental, by jumping or lying before moving object | No event |
| TN30.00 | Injury ?accidental, hanging | No event |
| TN3..00 | Injury ?accidental, hanging, strangulation and suffocation | No event |
| TN3z.00 | Injury ?accidental, hanging/strangulation/suffocation NOS | No event |
| TN3y.00 | Injury ?accidental, other means of hang/strangle/suffocate | No event |
| 8G61.00 | Potential suicide care | No event |
| ZX1R.00 | Throwing self in front of vehicle | High |

**Table S5: International Classification of Diseases-10th revision (ICD-10) codes of suicide by level of intent**

| **ICD-10** | **Term for ICD code** | **Intent level** |
| --- | --- | --- |
| X60 | Intentional self-poisoning by and exposure to nonopioid analgesics, antipyretics and antirheumatics | High |
| X61 | Intentional self-poisoning by and exposure to antiepileptic, sedative-hypnotic, antiparkinsonism and psychotropic drugs, not elsewhere classified | High |
| X62 | Intentional self-poisoning by and exposure to narcotics and psychodysleptics [hallucinogens], not elsewhere classified | High |
| X63 | Intentional self-poisoning by and exposure to other drugs acting on the autonomic nervous system | High |
| X64 | Intentional self-poisoning by and exposure to other and unspecified drugs, medicaments and biological substances | High |
| X65 | Intentional self-poisoning by and exposure to alcohol | Undefinable |
| X66 | Intentional self-poisoning by and exposure to organic solvents and halogenated hydrocarbons and their vapors | High |
| X67 | Intentional self-poisoning by and exposure to other gases and vapors | High |
| X68 | Intentional self-poisoning by and exposure to pesticides | High |
| X69 | Intentional self-poisoning by and exposure to other and unspecified chemicals and noxious substances | High |
| X70 | Intentional self-harm by hanging, strangulation and suffocation | High |
| X71 | Intentional self-harm by drowning and submersion | High |
| X72 | Intentional self-harm by handgun discharge | No event |
| X73 | Intentional self-harm by rifle, shotgun and larger firearm discharge | High |
| X74 | Intentional self-harm by other and unspecified firearm discharge | High |
| X75 | Intentional self-harm by explosive material | High |
| X76 | Intentional self-harm by smoke, fire and flames | High |
| X77 | Intentional self-harm by steam, hot vapors and hot objects | Undefinable |
| X78 | Intentional self-harm by sharp object | Undefinable |
| X79 | Intentional self-harm by blunt object | Undefinable |
| X80 | Intentional self-harm by jumping from a high place | High |
| X81 | Intentional self-harm by jumping or lying before moving object | High |
| X82 | Intentional self-harm by crashing of motor vehicle | High |
| X83 | Intentional self-harm by other specified means | Undefinable |
| X84 | Intentional self-harm by unspecified means | Undefinable |
| Y20 | Hanging, strangulation and suffocation, undetermined intent | Moderate |

**Table S6: Methods for identifying healthcare resource utilisation outcomes**

| **Outcome** | **Identification method*** |
| --- | --- |
| GP consultations | Records in CPRD consultation files. Consultation types (face-to-face, telephone, and other) were categorised using the approach in Kontopantelis et al. 2015^[[1]](#footnote-1)^. |
| IAPT services^†^ | MHDS non-inpatient mental health team episodes with clinical team type “Psychological Therapy Service (IAPT)” |
| Psychiatric outpatient services (CMHT or CRHT) ^†^ | Records of health care professional contacts in MHDS, with team type “Crisis Resolution Team/Home Treatment Service”, “Community Mental Health Team – Functional” or “Community Mental Health Team – Organic” |
| Psychiatrist visits^†^ | Records of Health Care Professional contacts in MHDS or records in HES outpatient, with main specialty “Adult mental illness”, “Child and adolescent psychiatry” or ”Old age psychiatry”. |
| A&E visits | Records in the HES Accident & Emergency attendance database. Only multi-specialty emergency departments with full resuscitation facilities and designated accommodation for A&E patients were considered (code 1 for department type). |
| Hospitalisations | Records in HES admissions with admission date within the period of interest. |
| Psychiatric inpatient services | Records of HES admissions with main specialty “Adult mental illness”, “Child and adolescent psychiatry” or ”Old age psychiatry”. Psychiatric episodes following each other with 0 days gaps were grouped together. |
| ICU admission | HES critical care records |
| PICU admission^†^ | Ward stay recorded in MHDS with intended clinical care intensity code 51 (“For Intensive Care - specially designated ward for patients needing containment and more intensive management (e.g. Psychiatric Intensive Care Unit (PICU)).“) |
| Single Point of Access | Records of health care professional contacts in MHDS, with team type “Single Point of Access Service” |
| 24/7 Crisis Response Line | Records of health care professional contacts in MHDS, with team type “24/7 Crisis Response Line” |
| Referral to CMHT | Records in referrals file corresponding to read terms “Referral to mental health team” (read code: 8Hc..00), ”Referral to community mental health team” (8Hc0.00),”Referral to non NHS mental health community service”(8HHn.00),”Referral to older age community mental health team”(8HHo.00) |
| Referral to improving access to psychological therapies (IAPT) | Records in referrals file corresponding to read term “Referral to improving access to psychological therapies prog” (read code: 8HkK.00). |
| Referral to Single Point of Access | Records in referrals file corresponding to read term “Referral to Single Point of Access service” (8T0Q.00) |

GP: General practitioner; IAPT: Improving access to psychological therapies; CMHT: Community Mental Health Team; CRHT: Crisis Resolution and Home Treatment Team); A&E: Accident & Emergency; PICU: Psychiatric Intensive Care Unit; ICU: Intensive Care Unit

*In case of duplicate visits at the same date, only one observation per patient, date, and main speciality, if available, was kept. When it was recorded that the patient did not attend the appointment, the visit was not counted.

^†^MHDS data used; this analysis includes only patients with relevant follow-up (4 weeks or 1 year) before 30/11/2015.

1. Kontopantelis, E., et al., Primary care consultation rates among people with and without severe mental illness: a UK cohort study using the Clinical Practice Research Datalink. BMJ open, 2015. 5(12). [↑](#footnote-ref-1)
